# Supplementary material for: Optimizing one-dose and two-dose cholera vaccine allocation in outbreak settings: A modeling study
Source: PLoS Negl Trop Dis. 2022 Apr 20;16(4):e0010358. doi: 10.1371/journal.pntd.0010358 (PMC9060364; doi:10.1371/journal.pntd.0010358)
Supplement: S1 Appendix — (PDF) [file pntd.0010358.s001.pdf]

Optimizing one-dose and two-dose cholera vaccine allocation in outbreak settings: A modeling study

Tiffany Leung[1], Julia Eaton[2], Laura Matrajt[1*],

1 Vaccine and Infectious Disease Division, Fred Hutchinson Cancer Research Center, Seattle, Washington, United States of America
2 School of Interdisciplinary Arts and Sciences, University of Washington, Tacoma, Washington, United States of America

* laurama@fredhutch.org

Appendix

The mathematical models

The urban city model: N'Djamena, Chad

We constructed a transmission model to simulate a cholera outbreak in an urban city and calibrated it to the 2011 cholera outbreak in N'Djamena, Chad (Fig A). Susceptible individuals (S) are infected through contact with an infectious individual or with a water source. Upon infection, the individual becomes exposed (E) where they are infected but not yet infectious for an average $1/\gamma_E$ days. A fraction k of these exposed individuals will experience symptoms and transition to the infectious with symptoms (I) class. The remaining fraction $(1 - k)$ will be asymptomatically infectious (A). Both infectious classes shed bacteria into the environment (water source). We assume that asymptomatic infectious individuals have a reduced degree of infectiousness b_A and reduced degree of bacterial shedding b_μ relative to symptomatic individuals. Natural immunity is assumed to last $1/\sigma$ (Erlang distributed) until the recovered individual becomes fully susceptible to infection again.

Environmental or waterborne transmission is measured by the amount of bacteria shed into the water reservoir (W). An infectious individual sheds bacteria in the water at rate μ, and bacteria decays at a rate δ. The force of infection is the sum of direct transmission by contact with an infectious individual and indirect transmission by contact with contaminated water, as measured by β and β_W respectively. Transmission by water is modulated by rainfall, which has been shown to be an important factor in cholera epidemics [1, 2]. A description of the model parameters is found in Table A.

The refugee camp model: Maela, Thailand

We extended a previously developed transmission model of cholera in Maela, the largest refugee camp in Thailand [3] (Fig B). The formulation of the model is explained in detail in the work of Havumaki et al. [3]. Whereas the original version of the model used an exponentially distributed duration of immunity, immunity waned over two recovered compartments leading to an Erlang distributed duration of immunity. A table of the model parameters is found in Table B.

The region torn by natural disaster model: Haiti

We used a previously developed meta-population model of cholera transmission in Haiti that consists of ten administrative departments [4] (Fig C). Details of the model are found in Lee et al. [4]. We made no changes to the transmission dynamics of this model. Model parameters are found in Table C.

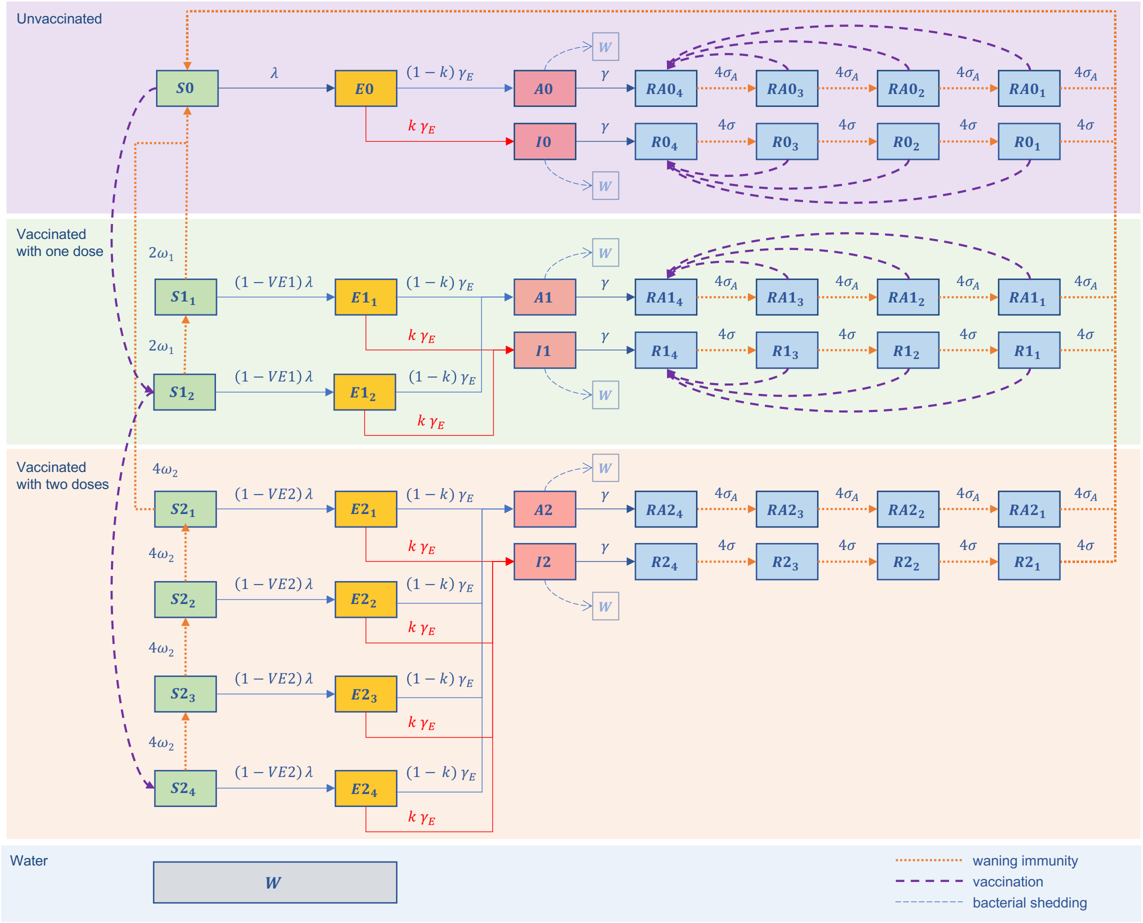

Fig A. Diagram of the model of cholera transmission and vaccination in Chad.

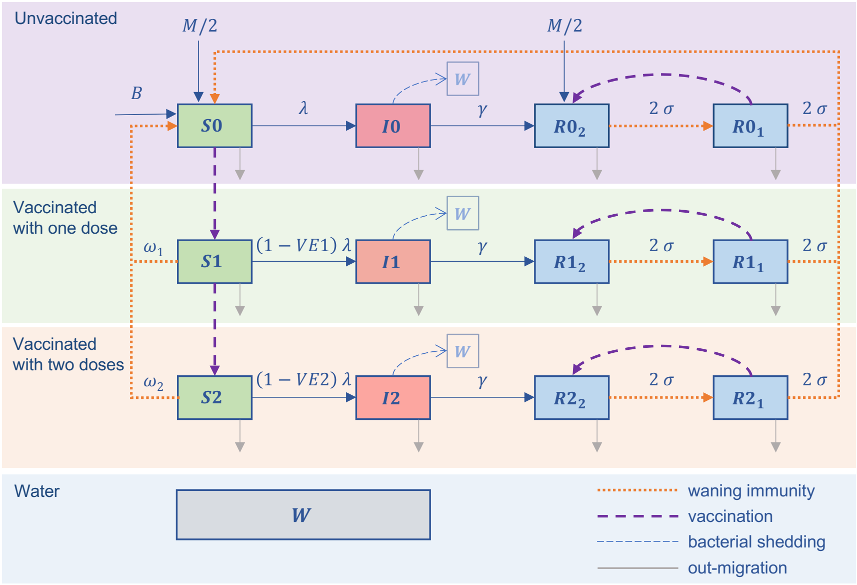

Fig B. Diagram of the model of cholera transmission and vaccination in Maela.

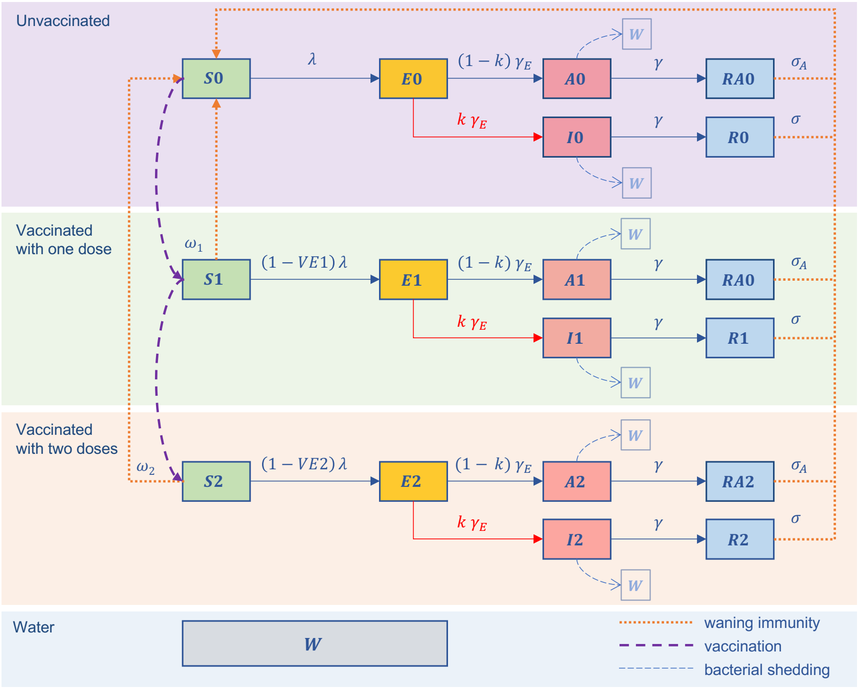

Fig C. Diagram of the model of cholera transmission and vaccination in Haiti.

Model Equations

The system of equations for the Chad model (age group omitted for clarity) are:
Unvaccinated:

$$\begin{aligned}
\frac{dS_0}{dt} &= \lambda S_0 + 4\sigma(RI_{0,1} + RI_{1,1} + RI_{2,1}) + 4\sigma_A(RA_{0,1} + RA_{1,1} + RA_{2,1}) + 2\omega_1 S_{1,1} + 4\omega_2 S_{2,1}\,, \\
\frac{dE_0}{dt} &= \lambda S_0 - \gamma_E E_0\,, \\
\frac{dI_0}{dt} &= k\gamma_E E_0 - \gamma I_0\,, \\
\frac{dA_0}{dt} &= (1-k)\gamma_E E_0 - \gamma A_0\,, \\
\frac{dRI_{0,4}}{dt} &= \gamma I_0 - 4\sigma RI_{0,4}\,, \\
\frac{dRI_{0,3}}{dt} &= 4\sigma RI_{0,4} - 4\sigma RI_{0,3}\,, \\
\frac{dRI_{0,2}}{dt} &= 4\sigma RI_{0,3} - 4\sigma RI_{0,2}\,, \\
\frac{dRI_{0,1}}{dt} &= 4\sigma RI_{0,2} - 4\sigma RI_{0,1}\,, \\
\frac{dRA_{0,4}}{dt} &= \gamma A_0 - 4\sigma RA_{0,4}\,, \\
\frac{dRA_{0,3}}{dt} &= 4\sigma RA_{0,4} - 4\sigma RA_{0,3}\,, \\
\frac{dRA_{0,2}}{dt} &= 4\sigma RA_{0,3} - 4\sigma RA_{0,2}\,, \\
\frac{dRA_{0,1}}{dt} &= 4\sigma RA_{0,2} - 4\sigma RA_{0,1}\,,
\end{aligned}$$

Vaccinated with one dose:

$$
\begin{aligned}
\frac{dS_{1,2}}{dt} &= -(1-VE1)\lambda S_{1,2} - 2\omega_1 S_{1,2}\,, \\
\frac{dS_{1,1}}{dt} &= -(1-VE1)\lambda S_{1,1} - 2\omega_1 S_{1,1} + 2\omega_1 S_{1,2}\,, \\
\frac{dE_{1,2}}{dt} &= (1-VE1)\lambda S_{1,2} - \gamma_E E_{1,2}\,, \\
\frac{dE_{1,1}}{dt} &= (1-VE1)\lambda S_{1,1} - \gamma_E E_{1,1}\,, \\
\frac{dI_1}{dt} &= k\gamma_E E_{1,2} + k\gamma_E E_{1,1} - \gamma I_1\,, \\
\frac{dA_1}{dt} &= (1-k)\gamma_E E_{1,2} + (1-k)\gamma_E E_{1,1} - \gamma A_1\,, \\
\frac{dRI_{1,4}}{dt} &= \gamma I_1 - 4\sigma RI_{1,4}\,, \\
\frac{dRI_{1,3}}{dt} &= 4\sigma RI_{1,4} - 4\sigma RI_{1,3}\,, \\
\frac{dRI_{1,2}}{dt} &= 4\sigma RI_{1,3} - 4\sigma RI_{1,2}\,, \\
\frac{dRI_{1,1}}{dt} &= 4\sigma RI_{1,2} - 4\sigma RI_{1,1}\,, \\
\frac{dRA_{1,4}}{dt} &= \gamma A_1 - 4\sigma_A RA_{1,4}\,, \\
\frac{dRA_{1,3}}{dt} &= 4\sigma RA_{1,4} - 4\sigma_A RA_{1,3}\,, \\
\frac{dRA_{1,2}}{dt} &= 4\sigma RA_{1,3} - 4\sigma_A RA_{1,2}\,, \\
\frac{dRA_{1,1}}{dt} &= 4\sigma RA_{1,2} - 4\sigma_A RA_{1,1}\,,
\end{aligned}
$$

Vaccinated with two doses:

$$\begin{aligned}
\frac{dS_{2,4}}{dt} &= -(1-VE2)\lambda S_{2,4} - 4\omega_2 S_{2,4}\,, \\
\frac{dS_{2,3}}{dt} &= -(1-VE2)\lambda S_{2,3} - 4\omega_2 S_{2,3} + 4\omega_1 S_{2,4}\,, \\
\frac{dS_{2,2}}{dt} &= -(1-VE2)\lambda S_{2,2} - 4\omega_2 S_{2,2} + 4\omega_1 S_{2,3}\,, \\
\frac{dS_{2,1}}{dt} &= -(1-VE2)\lambda S_{2,1} - 4\omega_2 S_{2,1} + 4\omega_1 S_{2,2}\,, \\
\frac{dE_{2,4}}{dt} &= (1-VE2)\lambda S_{2,4} - \gamma_E E_{2,4}\,, \\
\frac{dE_{2,3}}{dt} &= (1-VE2)\lambda S_{2,3} - \gamma_E E_{2,3}\,, \\
\frac{dE_{2,2}}{dt} &= (1-VE2)\lambda S_{2,2} - \gamma_E E_{2,2}\,, \\
\frac{dE_{2,1}}{dt} &= (1-VE2)\lambda S_{2,1} - \gamma_E E_{2,1}\,, \\
\frac{dI_2}{dt} &= k\gamma_E E_{2,4} + k\gamma_E E_{2,3} + k\gamma_E E_{2,1} + k\gamma_E E_{2,1} - \gamma I_2\,, \\
\frac{dA_2}{dt} &= (1-k)\gamma_E E_{2,4} + (1-k)\gamma_E E_{2,3} + (1-k)\gamma_E E_{2,2} + (1-k)\gamma_E E_{2,1} - \gamma A_2\,, \\
\frac{dRI_{2,4}}{dt} &= \gamma I_2 - 4\sigma RI_{2,4}\,, \\
\frac{dRI_{2,3}}{dt} &= 4\sigma RI_{2,4} - 4\sigma RI_{2,3}\,, \\
\frac{dRI_{2,2}}{dt} &= 4\sigma RI_{2,3} - 4\sigma RI_{2,2}\,, \\
\frac{dRI_{2,1}}{dt} &= 4\sigma RI_{2,2} - 4\sigma RI_{2,1}\,, \\
\frac{dRA_{2,4}}{dt} &= \gamma A_2 - 4\sigma_A RA_{2,4}\,, \\
\frac{dRA_{2,3}}{dt} &= 4\sigma RA_{2,4} - 4\sigma_A RA_{2,3}\,, \\
\frac{dRA_{2,2}}{dt} &= 4\sigma RA_{2,3} - 4\sigma_A RA_{2,2}\,, \\
\frac{dRA_{2,1}}{dt} &= 4\sigma RA_{2,2} - 4\sigma_A RA_{2,1}\,,
\end{aligned}$$

Water:

$$\frac{dW}{dt} = \mu_I(I_0 + I_1 + I_2) + \mu_A(A_0 + A_1 + A_2) - \delta W\,.$$

The force of infection λ is given by

$$\lambda = F(t)(\beta_W W)/(V+W) + [\beta(I_0 + I_1 + I_2) + b_A\beta(A_0 + A_1 + A_2)]\,,$$

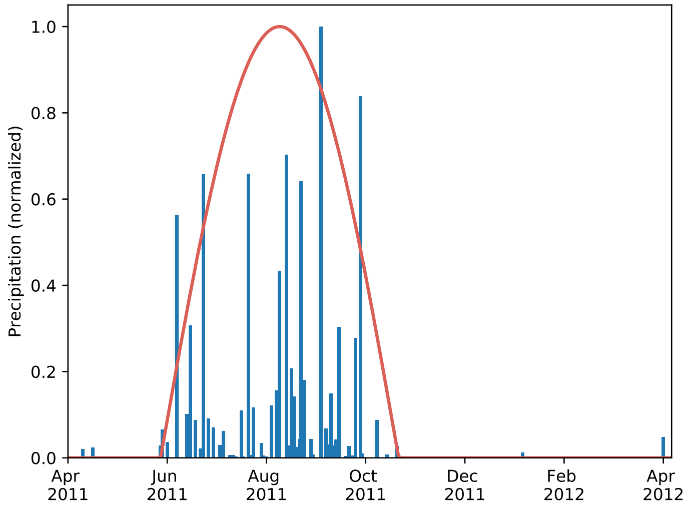

Fig D. Representation of the rainfall in Chad. Environmental transmission is modulated by a constant during the dry season (December to June) and by a sinusoidal function during the rainy season (June to November).

where

$$F(t) = \begin{cases} 0.9 \times \sin\left[\dfrac{\pi(T-56)}{200-56}\right] + 0.1, & \text{if } 56 \leq T \leq 200 \\ 0.1, & \text{otherwise,} \end{cases}$$

$T = (t - 365n)$ and the discrete year $n = (0, 1, 2, ...)$. The model diagram is found in Fig A.

We obtained daily precipitation data for N'Djamena from April 2011 to April 2012 from the Tropical Rainfall Measuring Mission Multi-satellite Precipitation Analysis (Version 7) [5][1]. During the rainy season between June and October, we modeled rainfall with a sine function (Fig D).

Model calibration: Chad

Daily incidence data for the 2011 cholera outbreak in N'Djamena, Chad were collected by Médecins Sans Frontières, and its details are found in [6]. The data spanned 232 days from July to November 2011. We fitted the model of Chad without age structure or vaccination using particle swarm optimization (pyswarms package in Python) [7, 8]). All model parameters were fixed except for four: person-to-person transmission, environment-to-person transmission, reporting ratio, and the number of recovered individuals at the start of simulation. In order to focus on the exponential phase of the outbreak, we fit the model from Day 100 of the data when the new cases increased exponentially. We assumed the cases from the first 100 days of the epidemic (1551 cumulative cases) had recovered and used that as a lower bound for number of recovered individuals at the

[1] `http://iridl.ldeo.columbia.edu/SOURCES/.NASA/.GES-DAAC/.TRMM_L3/.TRMM_3B42/.v7/.daily/.precipitation/X/15.0/15.25/RANGEEDGES/Y/12/12.25/RANGEEDGES/T/(01%20Apr%202011)(01%20May%202012)RANGEEDGES/`, accessed on March 10, 2019

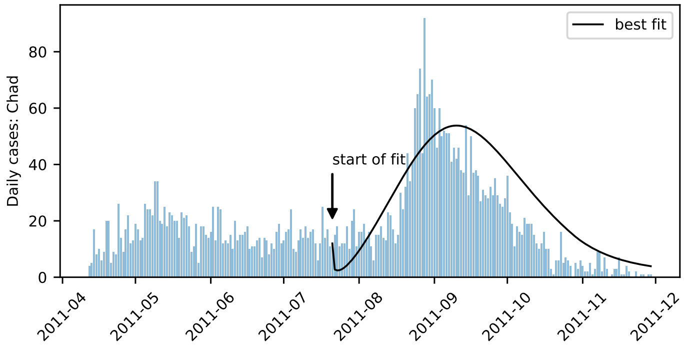

Fig E. Incidence data from Chad and the model fit without vaccination.

start of simulation. The calibrated model reproduced the epidemic curve of the 2011 epidemic in Chad (Fig E). All parameter values, including the fitted ones, are shown in Table A for Chad.

Vaccination dynamics

For each objective, the optimal allocation strategy determines how many doses to allocate to the two age groups that comprise the population (children under 5 years old, and those at least 5 years old). In the meta-population model of Haiti comprising ten administrative departments, we assume that the number of doses allocated per department is proportional to the population size of that department.

For a given optimal vaccine allocation strategy, the vaccination campaign is implemented in the following way. First, every individual allocated at least one dose receives a dose of vaccine. Then, those who are allocated two doses are vaccinated with their second dose. If the proposed number of vaccines allocated to an age group exceeded the population size of that group, the extra vaccines are assumed to be wasted.

In the vaccination campaign, available vaccine doses are distributed among susceptible and recovered individuals in proportion to their population size (pro-rata). Where the duration of immunity was Erlang-distributed (in Chad and Maela), recovered individuals who receive a vaccine have their immunity boosted. Where duration of immunity was exponentially distributed (in Haiti), only susceptible individuals were vaccinated. After an individual recovers from infection, immunity may wane.

Uncertainty Analysis

We assessed the uncertainty in the output measures of disease burden (percentage of cumulative infections averted, percentage of cumulative symptomatic infections averted, and percentage of cumulative deaths averted) by performing 1,000 simulations each with different parameter sets. Because each model had different features, the parameters that varied for this part of the analysis also differed. For Chad and Haiti, we sampled pre-determined gamma distributions with means given in Table A and C for the mean latent period and the mean infectious period. For the direct transmission coefficient, environmental transmission coefficient, relative infectiousness of asymp-

tomatic individuals, relative shedding of asymptomatic individuals, and fraction of infections that are symptomatic, we used truncated normal distributions with means and ranges given in Table A and C. For Maela, we sampled the infectious period from gamma distributions with means provided in Table B; the direct and environmental transmission coefficients and the reporting ratio for those at least five years old from truncated normal distributions with means and ranges specified in Table B. The reporting ratio for those under five years old was calculated with a multiplier (1.37 times higher). The shaded areas presented in the figures were obtained by removing the top and bottom 2.5% of the simulations.

Optimization

We developed a heuristic optimization procedure consisting of two stages allowing us to explore the entire vaccine space [9, 10]). We give a brief description here. The decision variable for the optimization problem is the proportion of the total vaccine available allocated to each of four groups (two age groups receiving one or two doses):

$$\sum_{i=1}^{4} f_i = 1,$$

where $f_i \geq 0$ is the fraction of the total available vaccine allocated to group i. The optimization procedure is as follows. In the first stage, we computed a mesh on the feasible set in 5% increments. We evaluated the metric (objective function) at each point on this mesh, to find the N best points (that is, those points with the N smallest metric values). In the second stage, we then randomly sampled N additional points from the Dirichlet distribution [11]. We use the $2N$ points as initial points for an unconstrained optimization method (Nelder-Mead [12, 13] or Particle Swarms [7, 8]), plus four additional starting points: one-dose-pro-rata, two-dose-pro-rata, one-dose-over-five, and the mixed strategy. Within the function evaluation step of the unconstrained method, the decision variable is corrected to satisfy $f_i \geq 0$ for $i = 1, \ldots, 4$ and $\sum_{i=1}^{4} f_i \leq 1$. The "optimal solution" is declared to be the best of these solutions.

Parameter	Meaning	Value (Range) [Units]	Source
**Chad:**			
β	direct transmission coefficient	3.31×10^{-6} (2.48×10^{-6}, 4.13×10^{-6}) [$\frac{1}{\text{p t}}$]	fitted
β_W	environmental transmission coefficient	1.43×10^{-3} (1.07×10^{-3}, 1.79×10^{-3}) [$\frac{1}{\text{p t}}$]	fitted
$1/\delta$	bacterial decay rate	21 [t]	[14]
$1/\gamma_E$	mean latent period	2 [t]	[15]
μ_I	bacterial shedding rate of symptomatic individuals	575 [$\frac{\text{cells/mL}}{\text{p t}}$]	
μ_A	bacterial shedding rate of asymptomatic individuals	0.01×575 [$\frac{\text{cells/mL}}{\text{p t}}$]	[15]
b_A	reduced infectiousness of asymptomatic infections	0.01 (0.0075, 0.0125)	[15]
b_μ	reduced bacterial shedding of asymptomatic infections	0.01 (0.0075, 0.0125)	[15]
$1/\sigma$	mean duration of natural immunity	4×365 [t]	[16, 17]
r	reporting ratio	0.08987	fitted
k	proportion of infections that are symptomatic	0.1 (0.05, 0.15)	[18, 19]
$1/\gamma$	mean infectious period	4 (2, 6) [t]	[6]
V	bacteria concentration yielding 50% of catching infection	10,000 [cells/mL]	[14]
$R(0)$	recovered individuals at the start of simulation	198,015 [p]	fitted
p_{under5}	proportion of population under 5 years old	0.193	[20]

Table A. Description of parameters used in the model of Chad, unless otherwise specified. (p = person; t = days)

Parameter	Meaning	Value (Range) [Units]	Source
**Maela:**			
β	direct transmission coefficient	7.4×10^{-6} $(5.6 \times 10^{-6}, 9.3 \times 10^{-6})$ $[\frac{1}{\text{p t}}]$	[3]
β_W	environmental transmission coefficient	6.3×10^{-7} $(4.7 \times 10^{-7}, 7.8 \times 10^{-7})$ $[\frac{1}{\text{p t}}]$	[3]
$1/\delta$	bacterial decay rate	62.5 [t]	[3]
μ	bacterial shedding rate	1	[3]
$1/\sigma$	mean duration of natural immunity	4×365 [t]	[16, 17]
r_{under5}	reporting ratio of those under 5 years old	0.019	[3]
r_{over5}	reporting ratio of those at least 5 years old	0.014 (0.0069, 0.0208)	[3]
$1/\gamma$	mean infectious period	4 (3.3, 4.7) [t]	[3]
N	total population	45,233 [p]	[3]
p_{under5}	proportion of population under 5 years old	0.132	[21]
d_{under5}	natural death rate for those under 5 years old	1.5×10^{-4} $[\frac{1}{\text{t}}]$	[3]
d_{over5}	natural death rate for those at least 5 years old	1.7×10^{-4} $[\frac{1}{\text{t}}]$	[3]
B	birth rate	2.8 $[\frac{\text{p}}{\text{t}}]$	[3]
M_{under5}	migration rate for susceptible or recovered individuals under 5 years old	1.1 $[\frac{\text{p}}{\text{t}}]$	[3]
M_{over5}	migration rate for susceptible or recovered individuals at least 5 years old	1.4 $[\frac{\text{p}}{\text{t}}]$	[3]

Table B. Description of parameters used in the model of Maela, including the values used, unless otherwise specified. (p = person; t = days)

Parameter	Meaning	Value (Range) [Units]	Source
**Haiti:**			
β	direct transmission coefficient	9.9×10^{-7} $(7.45 \times 10^{-7}, 1.24 \times 10^{-6})$ $[\frac{1}{\text{p t}}]$	[4]
β_W	environmental transmission coefficient	0.04 (0.03, 0.05) $[\frac{1}{\text{p t}}]$	[4]
$1/\delta$	bacterial decay rate	3 [t]	[4]
$1/\gamma_E$	mean latent period	0.18 (0.014, 0.550) [t]	[4]
μ_I	bacterial shedding rate of symptomatic individuals	3982.08 $[\frac{\text{cells/mL}}{\text{p t}}]$	[4]
μ_A	bacterial shedding rate of asymptomatic individuals	$10^{-7} \times \mu$ $[\frac{\text{cells/mL}}{\text{p t}}]$	[4]
b_A	relative infectiousness of asymptomatic infections	0.001 (0.00075, 0.00125)	[4]
b_μ	relative bacterial shedding of asymptomatic individuals	10^{-7} $(7.5{\times}10^{-7}, 1.25{\times}10^{-7})$	[4]
$1/\sigma$	mean duration of natural immunity	4×52 [t]	[16, 17]
r	reporting ratio	0.2	[4]
k	proportion of infections that are symptomatic	0.2 (0.1, 0.3)	[4]
$1/\gamma$	mean infectious period	1 (0.5, 1.8) [t]	[4]
V	bacteria concentration yielding 50% of catching infection	10^{-5} [cells/mL^3]	[4]
N	total population	10,911,819 [p]	[4]
p_{under5}	proportion of population under 5 years old	0.118	[4]

Table C. Description of parameters used in the model of Haiti, unless otherwise specified. (p = person; t = weeks)

References

[1] Moore SM, Azman AS, Zaitchik BF, Mintz ED, Brunkard J, Legros D, et al. El Niño and the shifting geography of cholera in Africa. Proceedings of the National Academy of Sciences of the United States of America. 2017;114(17):4436–4441.

[2] Koelle K, Rodó X, Pascual M, Yunus M, Mostafa G. Refractory periods and climate forcing in cholera dynamics. Nature. 2005;436(7051):696–700.

[3] Havumaki J, Meza R, Phares CR, Date K, Eisenberg MC. Comparing alternative cholera vaccination strategies in Maela refugee camp: Using a transmission model in public health practice. BMC Infectious Diseases. 2019;19(1):1–17.

[4] Lee EC, Chao DL, Lemaitre J, Matrajt L, Pasetto D, Perez-Saez J, et al. Achieving coordinated national immunity and cholera elimination in Haiti through vaccination. Lancet Global Health. 2020;8:e1081–1089.

[5] Huffman GJ, Adler RF, Bolvin DT, Nelkin EJ. The TRMM multi-satellite precipitation analysis (TMPA). Satellite Rainfall Applications for Surface Hydrology. 2008:1–19.

[6] Finger F, Bertuzzo E, Luquero FJ, Naibei N, Touré B, Allan M, et al. The potential impact of case-area targeted interventions in response to cholera outbreaks: A modeling study. PLoS Medicine. 2018;15(2):1–27.

[7] Kennedy J, Eberhart R. Particle swarm optimization. Proceedings of IEEE International Conference on Neural Networks. 1995;4:1942–1948.

[8] Miranda LJV. PySwarms: a research toolkit for Particle Swarm Optimization in Python. The Journal of Open Source Software. 2018;3(21):433.

[9] Matrajt L, Eaton J, Leung T, Brown ER. Vaccine optimization for COVID-19: Who to vaccinate first? Science Advances. 2021;7(6):eabf1374. Available from: http://advances.sciencemag.org/content/7/6/eabf1374.

[10] Matrajt L, Eaton J, Leung T, Dimitrov D, Schiffer JT, Swan DA, et al. Optimizing vaccine allocation for COVID-19 vaccines shows the potential role of single-dose vaccination. Nature Communications. 2021;12(3449). Available from: http://dx.doi.org/10.1038/s41467-021-23761-1.

[11] Kotz S, Balakrishnan N, Johnson NL. Continuous Multivariate Distributions. Volume 1: Models and Applications. Wiley; 2000.

[12] Nelder JA, Mead R. A simplex method for function minimization. The Computer Journal. 1965;7(4):308–313.

[13] Gao F, Han L. Implementing the Nelder-Mead simplex algorithm with adaptive parameters. Computational Optimization and Applications. 2012;51(1):259–277.

[14] Fung ICH. Cholera transmission dynamic models for public health practitioners. Emerging Themes in Epidemiology. 2014;11(1).

[15] Nelson EJ, Harris JB, Morris JG, Calderwood SB, Camilli A. Cholera transmission: The host, pathogen and bacteriophage dynamic. Nature Reviews Microbiology. 2009;7(10):693–702.

[16] Ali M, Emch M, Park JK, Yunus M, Clemens J. Natural cholera infection-derived immunity in an endemic setting. Journal of Infectious Diseases. 2011;204(6):912–918.

[17] Leung T, Matrajt L. Protection afforded by previous Vibrio cholerae infection against subsequent disease and infection: A review. PLOS Neglected Tropical Diseases. 2021;15(5):e0009383. Available from: `http://dx.doi.org/10.1371/journal.pntd.0009383`.

[18] Kaper JB, Morris Jr JG, Levine MM. Cholera. Clinical Microbiology Reviews. 1995;8(1):48–86.

[19] Jackson BR, Talkington DF, Pruckler JM, Fouché MDB, Lafosse E, Nygren B, et al. Seroepidemiologic survey of epidemic cholera in Haiti to assess spectrum of illness and risk factors for severe disease. American Journal of Tropical Medicine and Hygiene. 2013;89(4):654–664.

[20] Population Pyramid. Population of Chad 2011; 2021. Available from: `https://www.populationpyramid.net/chad/2011/`.

[21] Phares CR, Date K, Travers P, Déglise C, Wongjindanon N, Ortega L, et al. Mass vaccination with a two-dose oral cholera vaccine in a long-standing refugee camp, Thailand. Vaccine. 2016;34(1):128–133.
