# Supplementary figures and images for: Optimizing one-dose and two-dose cholera vaccine allocation in outbreak settings: A modeling study
Source: PLoS Negl Trop Dis. 2022 Apr 20;16(4):e0010358. doi: 10.1371/journal.pntd.0010358 (PMC9060364; doi:10.1371/journal.pntd.0010358)

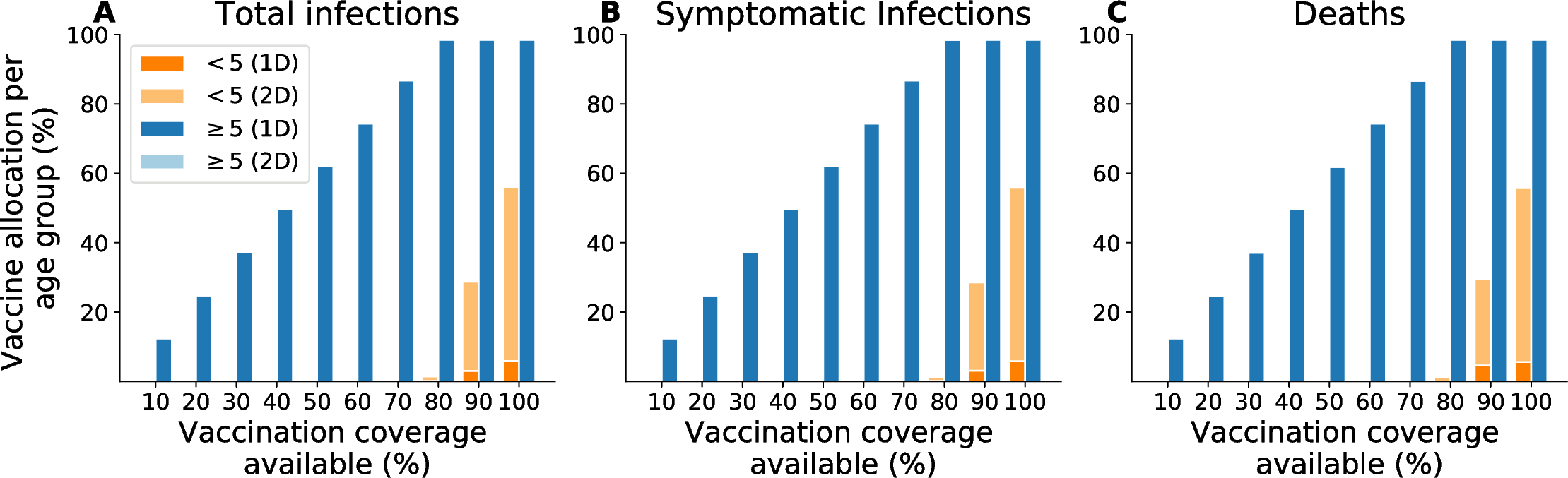

Supplement: S1 Fig — A: total infections. B: symptomatic infections. C: deaths. We considered enough vaccine to cover 10–100% of the population with a single dose. (TIF) [file pntd.0010358.s002.tif]

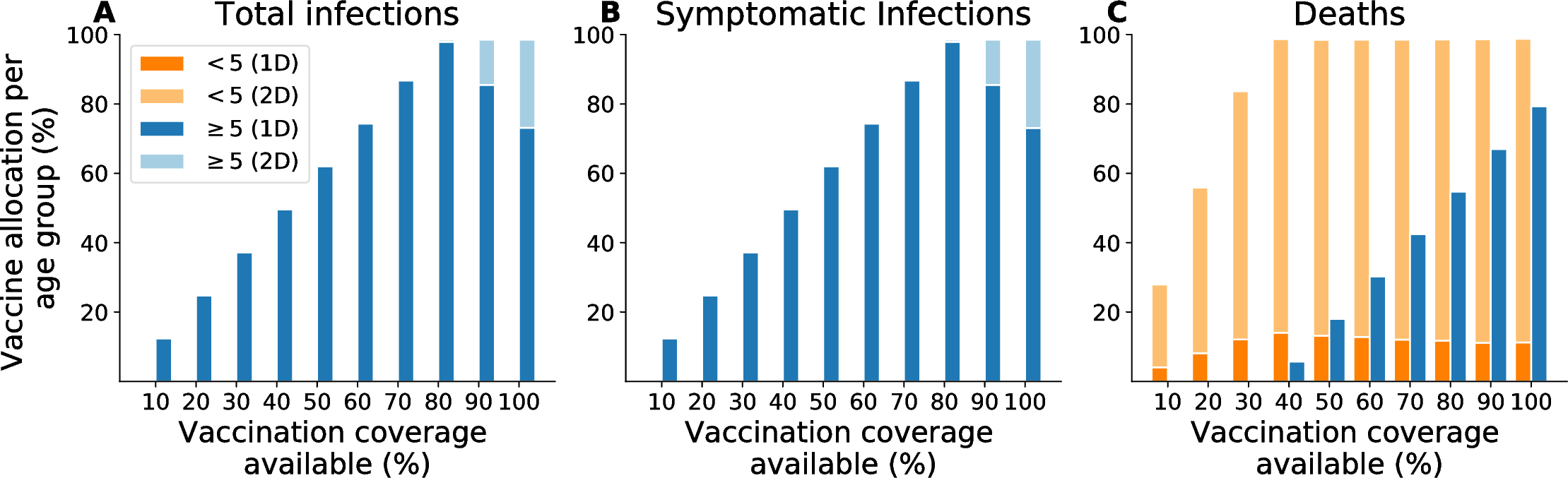

Supplement: S2 Fig — A: total infections. B: symptomatic infections. C: deaths. We considered enough vaccine to cover 10–100% of the population with a single dose. (TIF) [file pntd.0010358.s003.tif]

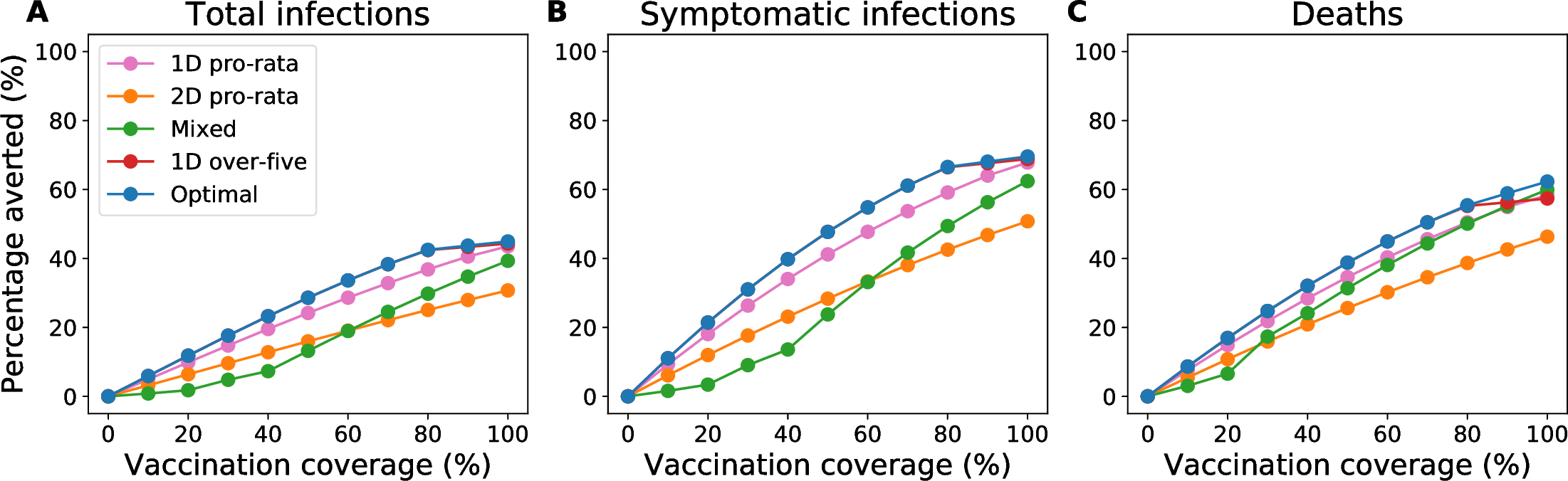

Supplement: S3 Fig — We considered enough vaccine to cover 10–100% of the population with a single dose. (TIF) [file pntd.0010358.s004.tif]

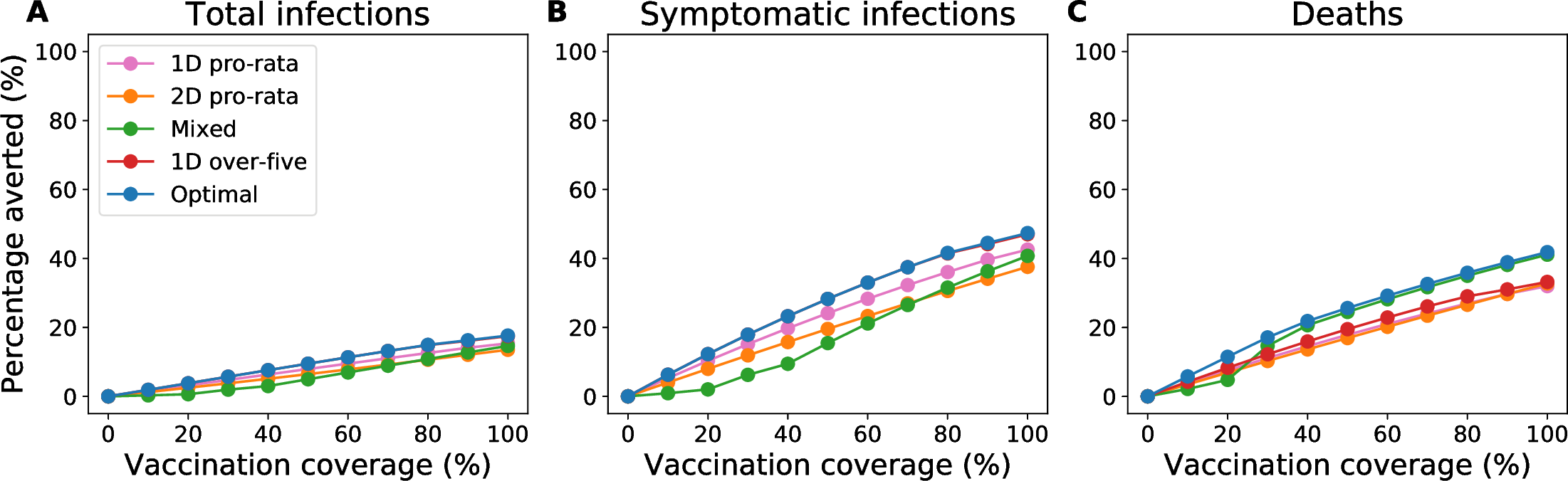

Supplement: S4 Fig — We considered enough vaccine to cover 10–100% of the population with a single dose. (TIF) [file pntd.0010358.s005.tif]
