# Supplementary material for: Optimizing one-dose and two-dose cholera vaccine allocation in outbreak settings: A modeling study
Source: PLoS Negl Trop Dis. 2022 Apr 20;16(4):e0010358. doi: 10.1371/journal.pntd.0010358 (PMC9060364; doi:10.1371/journal.pntd.0010358)
Supplement: S1 Table — (PDF) [file pntd.0010358.s006.pdf]

Optimizing one-dose and two-dose cholera vaccine allocation in outbreak settings: A modeling study

Tiffany Leung[1], Julia Eaton[2], Laura Matrajt[1*],

1 Vaccine and Infectious Disease Division, Fred Hutchinson Cancer Research Center, Seattle, Washington, United States of America
2 School of Interdisciplinary Arts and Sciences, University of Washington, Tacoma, Washington, United States of America

* laurama@fredhutch.org

Supplemental Table

Parameter	Meaning	Value < 5	Value ≥ 5	Source
**Vaccine efficacy:**				
$VE1$	reduced susceptibility to infection after 1 dose	0.08	0.575	[1, 2]
$VE2$	reduced susceptibility to infection after 2 doses	0.42	0.72	[3]
$1/\omega_1$	mean duration of immunity from 1 dose	2 years	2 years	[4]
$1/\omega_2$	mean duration of immunity from 2 doses	4 years	4 years	[5, 4, 3]
**Case fatality ratio:**				
	Chad:	0.21014	0.038	[6, 7]
	Thailand:	0.04977	0.009	[6, 8]
	Haiti:	0.07742	0.014	[9]

Table S1. Description of parameters related to the vaccine and disease metrics.

References

[1] Qadri F, Wierzba TF, Ali M, Chowdhury F, Khan AI, Saha A, et al. Efficacy of a single-dose, inactivated oral cholera vaccine in Bangladesh. New England Journal of Medicine. 2016;374(18):1723–1732.

[2] Qadri F, Ali M, Lynch J, Chowdhury F, Khan AI, Wierzba TF, et al. Efficacy of a single-dose regimen of inactivated whole-cell oral cholera vaccine: results from 2 years of follow-up of a randomised trial. The Lancet Infectious Diseases. 2018;18(6):666–674. Available from: `http://dx.doi.org/10.1016/S1473-3099(18)30108-7`.

[3] Bhattacharya SK, Sur D, Ali M, Kanungo S, You YA, Manna B, et al. 5 year efficacy of a bivalent killed whole-cell oral cholera vaccine in Kolkata, India: A cluster-randomised, double-blind, placebo-controlled trial. The Lancet Infectious Diseases. 2013;13(12):1050–1056.

[4] Franke MF, Ternier R, Jerome JG, Matias WR, Harris JB, Ivers LC. Long-term effectiveness of one and two doses of a killed, bivalent, whole-cell oral cholera vaccine in Haiti: an extended case-control study. The Lancet Global Health. 2018;6(9):e1028–e1035. Available from: `http://dx.doi.org/10.1016/S2214-109X(18)30284-5`.

[5] Bi Q, Ferreras E, Pezzoli L, Legros D, Ivers LC, Date K, et al. Protection against cholera from killed whole-cell oral cholera vaccines: A systematic review and meta-analysis. The Lancet Infectious Diseases. 2017;17(10):1080–1088.

[6] Troeger C, Blacker BF, Khalil IA, Rao PC, Cao S, Zimsen SR, et al. Estimates of the global, regional, and national morbidity, mortality, and aetiologies of diarrhoea in 195 countries: a systematic analysis for the Global Burden of Disease Study 2016. The Lancet Infectious Diseases. 2018;18(11):1211–1228.

[7] Ali M, Nelson AR, Lopez AL, Sack DA. Updated global burden of cholera in endemic countries. PLOS Neglected Tropical Diseases. 2015;9(6):1–13.

[8] Shannon K, Hast M, Azman AS, Legros D, McKay H, Lessler J. Cholera prevention and control in refugee settings: Successes and continued challenges. PLoS Neglected Tropical Diseases. 2019;13(6):1–11.

[9] Barzilay EJ, Schaad N, Magloire R, Mung KS, Boncy J, Dahourou GA, et al. Cholera surveillance during the Haiti epidemic — The first 2 years. New England Journal of Medicine. 2013;368(7):599–609.
