# Supplementary material for: Optimizing one-dose and two-dose cholera vaccine allocation in outbreak settings: A modeling study
Source: PLoS Negl Trop Dis. 2022 Apr 20;16(4):e0010358. doi: 10.1371/journal.pntd.0010358 (PMC9060364; doi:10.1371/journal.pntd.0010358)
Supplement: Attachment — Submitted filename: response_to_reviewers_2022_Leung.pdf [file pntd.0010358.s008.pdf]

Thank you for the opportunity to revise our manuscript PTND-D-21-01699, “Optimizing one-dose and two-dose cholera vaccine allocation in outbreak settings: A modeling study” for PLOS Neglected Tropical Diseases. We have addressed each reviewer’s comments (in blue) and describe our changes below in more detail.

Reviewer 1:

—-
This is an elegant modeling study evaluating different dosing strategies for using oral cholera vaccine. They use the cholera epidemiological patterns from three geographic areas in an attempt to capture different patterns in the different regions. These three areas have cholera patterns that are quite unique. I am assuming that other reviewers who are more skilled in modeling methods will provide additional review of the actual modeling mathematics, so my review will mainly deal with comments on how this model may be used for decision making.

It appears that the findings are quite consistent with earlier publications, especially “Azman AS, et al. The impact of a one-dose versus two-dose oral cholera vaccine regimen in outbreak settings: A modeling study. PLoS Medicine. 2015;12(8):e1001867.” This article which is cited previously concluded that a single dose strategy provides significant public heath benefit compared to two doses when the number of vaccine doses is limited in an outbreak situation, but it did not deal with more prolonged outbreaks or endemic situations which this new manuscript attempts to include.

The authors correctly point out that rapid deployment of vaccine is key to a successful vaccine campaign during outbreaks. Unfortunately, when attempting to vaccinate quickly, the current procedures for obtaining vaccine from a central global stockpile, even though being designed to be rapid, in fact often result in vaccinations beginning delivered well after the peak of the outbreak and sometimes even after the outbreak is over (see “Bwire G, et al. Use of surveys to evaluate an integrated oral cholera vaccine campaign in response to a cholera outbreak in Hoima district, Uganda. BMJ Open 2020, 10:e038464”. It would seem that, although the authors do stress this aspect, the role of delays in vaccination might be stressed even more and a sensitivity analysis to account for delays would be valuable.

Thank you very much for your positive comments about our work. In our manuscript, we considered allocating vaccine 15 and 115 days after the outbreak had started in Maela and Chad respectively, and 10 months following the devasting earthquake in 2010 (much longer waiting period) in Haiti. Our results show indeed that vaccination in Haiti, did not have as big an impact as it does in our other two other scenarios. We fully agree that delays to rapid OCV deployment should be stressed. We have expanded on this point in the discussion:

> *However, the procurement and deployment of OCV include significant delays. There is a median delay of three months between the declaration of an emergency and the start of the first round of vaccination [35]. Delay times range widely---the time from the first laboratory confirmation of cholera (or occurrence of humanitarian emergency) to the*

receipt of the official OCV request spans between 12 and 206 days [35]. These delays in vaccine shipment may result in campaigns starting after the outbreak is over [43].

The authors should describe the three mechanisms for accessing OCV from the global stockpile. These include 1) emergency use to control outbreaks, 2) emergency use to prevent outbreaks during a humanitarian crisis, and 3) preventive use of vaccine for areas determined to be hot-spots. It is not clear which of these situations apply to their model and the manuscript would benefit from aligning their model with these three.

For example, if one is using the vaccine for the emergency control of an outbreak, most outbreaks occur suddenly, and are of relatively short duration so nearly all the benefit occurs in the first year. Another outbreak may not occur for several years so determining a benefit over three years would show little impact relative to a one year impact and providing a second dose to this area will increase costs but provide little additional benefit. This would change of course, if this same area was also previously determined to be a hot-spot. (Outbreaks are more likely to occur in such hot-spots.)

If the campaign is intending to vaccinate a "hot-spot" where outbreaks occur frequently, the second dose could more readily demonstrate additional benefit. Both of these situations are also then different from the endemic situation in Bangladesh where cholera occurs continuously at predicable rates. Obviously, a model cannot consider every possible scenario, but the discussion could provide additional context for these different situations.

Thank you for the comments on OCV access from the global stockpile. We agree that it is beneficial to describe the three mechanisms for a request of OCV to give the reader additional context. We have added a few sentences in the introduction and in the discussion.

In the introduction:

> *The three main contexts under which countries may request doses from the global OCV stockpile include 1) emergency use to control an active outbreak, 2) emergency use to prevent outbreaks during a humanitarian crisis, and 3) preventive use for areas deemed ``hot-spots'' or where cholera is endemic [3].*

In the discussion:

> *It is important to consider that the optimal allocation of vaccine may change under different epidemiological contexts. In the present work we showed the optimal allocation of OCV under the context of emergency use to control an active outbreak (in epidemic situations). However, cholera outbreaks range widely depending on the region (short outbreaks are commonly seen in Africa while cholera is endemic in Bangladesh), and the optimal vaccine allocation may be different if used to prevent outbreaks or in endemic areas with hot spots.*

A specific queries and suggestions.

For the age group <5, did the model assume that all children under age 5 would be vaccinated or only the age group >1 year. (The vaccine is not approved for children <1 year).

We thank the reviewer for pointing out that this was not clear. Yes, the model assumed that all children under five years old were eligible to be vaccinated. We have now clarified this in the methods and added this as a limitation of the study:

> *We also made a simplifying assumption in our models that all children under five years old were eligible to be vaccinated. In reality, the vaccine is not approved for children under one year old.*

Reviewer's Responses to Questions

Reviewer #1: yes, the analysis matches the plan
The figures need to better label the x and y axes.

We thank the reviewer for this suggestion. We have updated the vertical axis label for the figures of the optimal vaccine allocation strategies and the figures of reductions in the metrics of disease burden to "Vaccination coverage ("% of total population)". We hope that this new label is more clear. Additionally we increased the font size of the ticks.

Reviewer 2:
—-
Overall comment:

1. Methods section is not clear and needs to be more specific.

We thank the reviewer for this comment. We have now modified the Methods section and hopefully improved its clarity.

We have made the following changes:

i) We have added a fuller description of the SEIRS-type model, which reads:

> *Susceptible (S) individuals are infected directly through contact with an infectious individual or indirectly through contact with contaminated water. They then become exposed but not yet infectious (E). After the latent period, they become infectious either with symptoms (I) or without symptoms (A). Eventually they recover (R) and are temporarily immune to infection until they become susceptible again. This model follows the general SEIRS framework, with two infectious compartments and a rainfall-modulated water reservoir (W) that tracks the concentration of V. cholerae (Figure 1A).*

ii) The general description of the models above now mentions waning immunity. This would reduce confusion to the reader when the durations of natural and vaccine-induced immunity are described later in the Methods. We have updated the model diagrams in Figure 1 by adding arrows for waning immunity as part of the general framework of the models. Additionally, we have elaborated on the epidemiological states in the figure caption. The Figure 1 caption now reads:

> *Figure 1: General framework of the models. The three models share common epidemiological states: susceptible (S), exposed but not yet infectious (E), infectious with symptoms (I), infectious without symptoms (A), and recovered (R). Infectious individuals shed bacteria into the water (W) (dashed line). Unique features of each model are shown in thick arrows. A: N'Djamena, Chad, a densely populated city. Transmission is influenced by rainfall. B: Maela, a refugee camp. Individuals may enter and exit the population through in- and out-migration. C: Haiti, where transmission is influenced by a road and water mobility network. These networks connect the ten departments. The age-structured and vaccinated analogues of these epidemiological states are omitted for clarity. See the SM for details.*

2. Inclusion and exclusion criteria needs to be better explained.

We are unsure what is meant by inclusion and exclusion criteria. If it meant the populations that are included in the vaccination campaign, then we have clarified that the entire population is eligible to receive vaccination (those aged 0 to 4 years, and those aged 5 years and older). We have listed in the limitations of the study that in reality the OCV is not approved for children under 1 year old.

3. In the discussion part, some comments:

   A. case-control (MCC), test-negative case-control (TNCC) and case-cohort study can be compared about effectiveness of one dose of oral cholera.

We agree that comments on vaccine effectiveness from different study types should be added. We have added the following to the discussion:

> *One-dose vaccine effectiveness from case-control; test-negative, case-control; and case-cohort studies range widely from 33% to 87%, and some of these estimates were not statistically significant [52, 12, 53, 17].*

   B. Besides influenza, comparison with other diarrhoeal agents including rotavirus needs to be made. In 2016, rotavirus infection caused more than 258 million episodes of diarrhea in children under the age of five.

We agree that comparison to rotavirus is important to add context to our results on cholera. We have included the following in the discussion:

> *Besides cholera, other diarrheal diseases pose significant burden. In 2016, rotavirus infection caused more than 258 million episodes of diarrhea among children under five years old globally [47]. Rotavirus vaccines are also delivered in multiple doses, and some studies have suggested that an incomplete series of the rotavirus vaccine may provide sufficient protection against severe disease and hospitalizations [48, 49, 50].*

Specific comments:

Page 5-9:

Result part: kindly specific about confounders.

We have added a few confounders to the limitation section of the paper. They now read:

> *Further studies are needed to truly evaluate the burden of cholera in young children. In particular, serological studies [52] might be useful to determine the burden of different diarrheal diseases in a particular region. We did not consider other possible confounders (e.g., differences in waning immunity and pre-existing immunity in endemic versus epidemic settings, the use of other interventions in tandem with vaccination) which could affect the population impact of vaccination.*

Page 11:

Better to add in the conclusion part the following
"However, in places where there was a lot of population migration, the long time between treatments meant that many people only got one OCV dose. In the event that the risk persists, more vaccination campaigns may be required to maintain protection over time." Or this can be added in the discussion part.

This is a very good point, and we thank the reviewer for this suggestion. The last sentence of the paper now reads:

> *However, in places where there is a lot of population migration, a long interval between the first and the second dose might result in having a proportion of the population receiving only one dose. In the event that the risk of future epidemics persist, recurrent vaccination campaigns may be required to maintain protection over time.*

Figure 1 : Elaboration of “S,E,A,I,R,W” is needed.

The caption of Figure 1 now elaborates on the SEIARW compartments to add more clarity to the Methods section. See our response to Reviewer 1’s comment above for what the caption now reads.
