# Supplementary material for: Optimizing one-dose and two-dose cholera vaccine allocation in outbreak settings: A modeling study
Source: PLoS Negl Trop Dis. 2022 Apr 20;16(4):e0010358. doi: 10.1371/journal.pntd.0010358 (PMC9060364; doi:10.1371/journal.pntd.0010358)
Supplement: Attachment — Submitted filename: PNTD_Reviewer_Comment.docx [file pntd.0010358.s007.docx]

**ID: PNTD-D-21-01699**

**Optimizing one-dose and two-dose cholera vaccine allocation in outbreak settings: A modeling study**

**Overall comment:**

1. Methods  section is not  clear and needs to be more specific.
2. Inclusion and exclusion criteria needs to be better explained.
3. In the discussion part, some comments:

A.      case-control (MCC), test-negative case-control (TNCC) and case-cohort study can be compared about effectiveness of one dose of oral cholera.

B.      Besides influenza, comparison with other diarrhoeal agents including rotavirus needs to be made. In 2016, rotavirus infection caused more than 258 million episodes of diarrhea in children under the age of five.

**Specific comments:**

*Page 5-9:*

Result part: kindly specific about confounders.

Page 11:

Better to add in the conclusion part the following

“However, in places where there was a lot of population migration, the long time between treatments meant that many people only got one OCV dose. In the event that the risk persists, more vaccination campaigns may be required to maintain protection over time.” Or this can be added in the discussion part.

Figure 1 : Elaboration of  “S,E,A,I,R,W” is needed.
